# Microbial Enzymes with Special Characteristics for Biotechnological Applications

**DOI:** 10.3390/biom3030597

**Published:** 2013-08-23

**Authors:** Poonam Singh Nigam

**Affiliations:** Biomedical Science Research Institute, University of Ulster, Coleraine BT52 1SA, UK; E-Mail: p.singh@ulster.ac.uk; Tel.: +44-28-7012-4053; Fax: +44-28-7012-4965

**Keywords:** microbial-enzymes, thermophilic, alkalophilic, thermostable, protease, keratinase, amylase, xylanase, laccase

## Abstract

This article overviews the enzymes produced by microorganisms, which have been extensively studied worldwide for their isolation, purification and characterization of their specific properties. Researchers have isolated specific microorganisms from extreme sources under extreme culture conditions, with the objective that such isolated microbes would possess the capability to bio-synthesize special enzymes. Various Bio-industries require enzymes possessing special characteristics for their applications in processing of substrates and raw materials. The microbial enzymes act as bio-catalysts to perform reactions in bio-processes in an economical and environmentally-friendly way as opposed to the use of chemical catalysts. The special characteristics of enzymes are exploited for their commercial interest and industrial applications, which include: thermotolerance, thermophilic nature, tolerance to a varied range of pH, stability of enzyme activity over a range of temperature and pH, and other harsh reaction conditions. Such enzymes have proven their utility in bio-industries such as food, leather, textiles, animal feed, and in bio-conversions and bio-remediations.

## 1. Enzymes from Microbial Sources

Enzymes are the bio-catalysts playing an important role in all stages of metabolism and biochemical reactions. Certain enzymes are of special interest and are utilized as organic catalysts in numerous processes on an industrial scale. Microbial enzymes are known to be superior enzymes obtained from different microorganisms, particularly for applications in industries on commercial scales. Though the enzymes were discovered from microorganisms in the 20th century, studies on their isolation, characterization of properties, production on bench-scale to pilot-scale and their application in bio-industry have continuously progressed, and the knowledge has regularly been updated. Many enzymes from microbial sources are already being used in various commercial processes. Selected microorganisms including bacteria, fungi and yeasts have been globally studied for the bio-synthesis of economically viable preparations of various enzymes for commercial applications [1]. 

In conventional catalytic reactions using biocatalysts the use of enzymes, either in free or in immobilized forms, is dependent on the specificity of enzyme. In recent advances of biotechnology, according to the requirements of a process, various enzymes have been and are being designed or purposely engineered. Various established classes of enzymes are specific to perform specialized catalytic reactions and have established their uses in selected bio-processes. A large number of new enzymes have been designed with the input of protein-engineering, biochemical-reaction engineering and metagenomics. Various molecular techniques have also been applied to improve the quality and performance of microbial enzymes for their wider applications in many industries [2]. As a result, many added-value products are being synthesized in global market with the use of established bioprocess-technology employing purposely engineered biocatalyst-enzymes.

Most of the commercially applicable proteases are alkaline and are bio-synthesized mainly by bacteria such as *Pseudomonas*, *Bacillus*, and *Clostridium*, and some fungi are also reported to produce these enzymes [3]. The xylanases with significant applications in bio-industries are produced by the fungal species belonging to genera *Trichoderma*, *Penicillium* and *Aspergillus*; the xylanases produced by these microorganisms have been found to possess high activity over a wide range of temperatures (40–60 °C) [4].

## 2. Enzymes with Special Characteristics

Special characteristics of microbial enzymes include their capability and appreciable activity under abnormal conditions, mainly of temperature and pH. Hence, certain microbial enzymes are categorized as thermophilic, acidophilic or alkalophilic. Microorganisms with systems of thermostable enzymes that can function at higher than normal reaction temperatures would decrease the possibility of microbial contamination in large scale industrial reactions of prolonged durations [5,6,7]. The quality of thermostability in enzymes promotes the breakdown and digestion of raw materials; also the higher reaction temperature enhances the penetration of enzymes [8]. The complete saccharification and hydrolysis of polysaccharides containing agricultural residues requires a longer reaction time, which is often associated with the contamination risks over a period of time. Therefore, the hydrolytic enzymes are well sought after, being active at higher temperatures as well as retaining stability over a prolonged period of processing at a range of temperatures. The high temperature enzymes also help in enhancing the mass-transfer and reduction of the substrate viscosity [9,10] during the progress of hydrolysis of substrates or raw materials in industrial processes. Thermophilic xylanase are considered to be of commercial interest in many industries particularly in the mashing process of brewing. The thermostable plant xerophytic isoforms of laccase enzyme are considered to be useful for their applications in textile, dyeing, pulping and bioremediation [1,4]. 

## 3. Enzymes with Special Characteristics in Biotechnology

### 3.1. Protease

Though the hydrolytic enzymes belong to the largest group of enzymes and are the most commercially-applicable enzymes, among the enzymes within this group the microbial proteases have been extensively studied [11,12,13,14,15,16]. Proteases prepared from microbial systems are of three types: acidic, neutral and alkaline. Alkaline proteases are efficient under alkaline pH conditions and consist of a serine residue at their active site [15]. Alkaline serine proteases have the largest applications in bio-industry. Alkaline proteases are of particular interest being more suitable for a wide range of applications, since these possess high activity and stability in abnormal conditions of extreme physiological parameters. Alkaline proteases have shown their capability to work under high pH, temperature and in presence of inhibitory compounds [15,16,17,18].

Vijayalakshmi *et al*. [16] have optimized and characterized the cultural conditions for the production of alkalophilic as well as a thermophilic extracellular protease enzyme from *Bacillus*. This bacteria named *Bacillus* RV.B2.90 was found to be capable of producing an enzyme preparation possessing special characteristics such as being highly alkalophilic, moderately halophilic, thermophilic, and exhibiting the quality of a thermostable protease enzyme. Alkaline proteases possess the property of a great stability in their enzyme activity when used in detergents [16,18,19]. The alkaline protease produced from *Bacilli* and proteases from other microorganisms have found more applications overall in bio-industries such as: washing powders, tannery, food-industry, leather processing, pharmaceuticals, for studies in molecular biology and in peptide synthesis [1,3].

### 3.2. Keratinases

Keratin is an insoluble and fibrous structural protein that is a constituent of feathers and wool. The protein is abundantly available as a by-product from keratinous wastes, representing a valuable source of proteins and amino acids that could be useful for animal feeds or as a source of nitrogen for plants [20]. However, the keratin-containing substrates and materials have high mechanical stability and hence are difficult to be degraded by common proteases. Keratinases are specific proteolytic enzymes which are capable of degrading insoluble keratins. The importance of these enzymes is being increasingly recognized in fields as diverse as animal feed production, textile processing, detergent formulation, leather manufacture, and medicine. Proteolytic enzymes with specialized keratinase activity are required to degrade keratins and for this purpose the keratinases have been isolated and purified from certain bacteria, actinomycetes, and fungi [20,21]. 

Keratinases have been classified as serine- or metallo-proteases. Cloning and expression of keratinase genes in a variety of expression systems have also been reported [22]. A higher operation temperature is required in the degradation of materials like feathers and wool, which would be possible using a thermostable keratinase. This aspect is of added advantage in achieving a higher reactivity due to lower diffusional restrictions and hence a higher reaction rate would be established. The enhanced stability of keratinase would increase the overall process yield due to the increased solubility of keratin and favorable equilibrium displacement in endothermic reactions.

Baihong *et al*. [23] have reported the enhanced thermostability of a preparation of keratinase by computational design and empirical mutation. The quadruple mutant of *Bacillus subtilis* has been characterised to exhibit the synergistic and additive effects at 60 °C with an increase of 8.6-fold in the t1/2 value. The N122Y substitution also led to an approximately 5.6-fold increase in catalytic efficiency compared to that of the wild-type keratinase.

An alkalophilic strain of *Streptomyces albidoflavus* has been reported to produce extracellular proteases [24]. This particular type of protease was capable of hydrolyzing keratin. The biosynthesis of this specific enzyme was optimized in submerged batch cultures at highly alkaline pH 10.5 and the enzyme yield was stimulated by using an inducer substrate containing keratin in the form of white chicken feathers. An enhanced (six-fold) protease production could be achieved with modified composition of culture-medium containing inducer at the concentration of 0.8% in the fermentation medium. The novelty of this crude enzyme has been reported to be its activity and stability in neutral and alkaline conditions. The maximum activity has been obtained at pH 9.0 and in the temperature range of 60–70 °C. This type of protease (keratinase- hydrolyzing keratins) is of particular significance for its application in industries since the crude enzyme showed its tolerance to the detergents and solvents tested [24]. Liu *et al*. [25] have studied the expression of extreme alkaline, oxidation-resistant keratinase from *Bacillus licheniformis* into the recombinant *Bacillus subtilis* WB600 expression system. The alkaline keratinase was characterized for its application in the processing of wool fibers. 

### 3.3. Amylase

Amylases are significant enzymes for their specific use in the industrial starch conversion process [26]. Amylolytic enzymes act on starch and related oligo- and polysaccharides [27]. The global research on starch hydrolyzing enzymes based on the DNA sequence, structural analysis and catalytic mechanism has led to the concept of one enzyme family—the alpha amylase. The amylolytic and related enzymes have been classified as glycoside hydrolases. The enzymes have been produced by a wide range of microorganisms and substrates [28,29,30] and categorized as exo-, endo-, de-branching and cyclodextrin producing enzyme. The application of these enzymes has been established in starch liquefaction, paper, food, sugar and pharmaceutical industries. In the food industry amylolytic enzymes have a large scale of applications, such as the production of glucose syrups, high fructose corn syrups, maltose syrup, reduction of viscosity of sugar syrups, reduction of turbidity to produce clarified fruit juice for longer shelf-life, solubilisation and saccharification of starch in the brewing industry [31]. The baking industry uses amylases to delay the staling of bread and other baked products; the paper industry uses amylases for the reduction of starch viscosity to achieve the appropriate coating of paper. Amylase enzyme is used in the textile industry for warp sizing of textile fibers, and used as a digestive aid in the pharmaceutical industry [28].

Li *et al*. [32] have recently isolated, characterized and cloned a themotolerant isoamylase. For this purpose the enzyme was bio-synthesized using a thermophilic bacterium *Bacillus* sp. This novel enzyme has been reported to display its optimal activity at a remarkably high temperature of 70 °C, as well as being active in the alkaline range. This thermophilic enzyme has also been found to be thermo-stable between 30 and 70 °C, and its activity has been reported to be stable within a pH range of 5.5 to 9.0. 

Gurumurthy *et al*. [33] completed the molecular characterization of an extremely thermostable alpha-amylase for industrial applications. This novel enzyme was produced by a bacterium *Geobacillus* sp which was isolated from the thermal water of a geothermal spring. This isolated bacterium showed the characteristics of thermo-tolerance and alkali-resistance. A purified preparation of amylase suitable for application was obtained using a DEAE-cellulose column and Sephadex G-150 gel filtration chromatography. The enzyme is a novel alpha-amylase due to its optimum activity at a very high temperature of 90 °C and an alkaline pH 8.0. However, this purified preparation enzyme was found to be stable only for 10 min at 90 °C. 

### 3.4. Xylanase

Hemicellulose is one of main constituents of agricultural residues and plants along with cellulose, lignin and pectin [34]. Xylan is the major component of hemicellulose consisting of β-1,4-linked D-xylopyranosyl residues. The hydrolysis of xylan in plant materials is achieved by the use of a mixture of hydrolytic enzymes including endo-β-1,4-xylanase and β-D-xylosidase [35]. The importance of xylanase has tremendously increased due to its biotechnological applications for pentose production, fruit-juice clarification, improving rumen digestion and the bioconversion of lignocellulosic agricultural residues to fuels and chemicals [34]. Collins *et al*. [36] have extensively studied the xylanase enzyme and its families as well as the special xylanases possessing extremophilic characteristics. Xylanases have established their uses in the food, pulp, paper and textile industries, agri-industrial residues utilization, and ethanol and animal feed production [37,38].

The enzyme used for the purpose of bio-bleaching of wood pulp should be active in the conditions of alkaline pH, high temperature and at the same time it is desirable that this enzyme is stable at high reaction temperatures. Xylanase preparations used for wood processing in the paper industry should be free of cellulose activity. Cellulase-free xylanase preparations have applications in the paper industry to provide brightness to the paper due to their preferential solubilisation of xylans in plant materials and selective removal of hemicelluloses from the kraft-pulp. Kohli *et al*. [39] have studied the production of a c cellulase free extracellular endo-1,4-β-xylanase at a higher temperature of 50 °C and at pH 8.5 employing a selected microorganism: *Thermoactinomyces thalophilus*. The enzyme preparation was found to be thermostable at 65 °C, retaining its activity at 50% after 125 min of incubation at 65 °C. The crude enzyme preparation showed no cellulase activity and the optimum temperature and pH for maximum xylanase activity was found to be 65 °C and 8.5–9.0, respectively. A thermotolerant and alkalotolerant xylanase has been reported to be produced by *Bacillus* sp [40]. To make the applications of xylanase viable on commercial scales, heterologous systems of *Escherichis coli*, *Pichia pastoris* and *Bacillus* sp have been used to express xylanase activity [41,42]. The thermophilic microorganism *Humicola* spp. has been studied for its capability of bio-synthesising an alkali-tolerant β-mannase xylanase [43]. Acidophilic xylanases stable under acidic conditions of reaction are reported to be produced by an acidophilic fungus *Bispora* [44], in contrast a xylanase active under conditions of alkaline pH has been studied by Mamo *et al*. [45] for the mechanism of their high pH catalytic adaptation. 

Recently three novel xylanases thermophilic in nature (XynA,B,C) have been characterized by Yanlong *et al*. [46], these were produced by *Humicola* sp. for their potential applications in the brewing industry. One xylanase gene, XynA, has been found to adapt to alkaline conditions and stability at higher temperatures. This XynA also possessed higher catalytic efficiency and specificity for a range of substrates. Yanlong *et al*. [46] have reported the application of three xylanases, XynA-C, in simulated mashing conditions in the brewing industry and found the better performance of 37% on filtration acceleration and 13% reduction in viscosity of substrate in comparison to the performance of a commercial trade enzyme, Ultraflo, a product from Novozyme. 

### 3.5. Laccase/Ligninase

Ligninolytic enzymes are applicable in the hydrolysis of lignocellulosic agricultural residues, particularly for the degradation of the complex and recalcitrant constituent lignin. This group of enzymes is a mixture of synergistic enzymes, hence they are highly versatile in nature and can be used in a range of industrial processes [47,48,49]. The complex enzyme system consists of three oxidative enzymes: lignin peroxidase (LiP), manganese peroxidase (MnP) and laccase. These enzymes have established their applications in bio-remediation, pollution control and in the treatment of industrial effluents containing recalcitrant and hazardous chemicals such as textile dyes, phenols and other xenobiotics [50,51,52,53].

The paper and pulp industry requires a step of separation and degradation of lignin from plant material, where the pretreatment of wood pulp using ligninolytic enzymes is important for a milder and cleaner strategy of lignin removal compared to chemical bleaching. Bleach enhancement of mixed wood pulp has been achieved using co-culture strategies, through the combined activity of xylanase and laccase [54]. The ligninolytic enzyme system is used in bio-bleaching of craft pulp and in other industries such as for the stabilization of wine and fruit juices, denim washing [49], the cosmetic industry and biosensors [1,34]. Fungi are the most potent producers of lignin degrading enzymes. White rot fungi have been specifically studied for the production of these enzymes by Robinson *et al*. [50,51,52]. For the economical production of ligninolytic enzymes, agricultural residues have been used as the substrate in microbial production of lignin degrading enzymes [34].

Thermophilic laccase enzyme is of particular use in the pulping industry. Recently, Gali and Kotteazeth [55] reported the biophysical characterization of thermophilic laccase isoforms. These were initially isolated from the xerophytic plant species *Cereus pterogonus* and *Opuntia vulgaris* and showed thermophilic property [56,57,58]. In order to prepare laccase enzymes with special characteristics, several studies have been conducted to provide a scientific basis for the employment of laccases in biotechnological processes [59,60,61,62]. Forms of laccase with unusual properties have been isolated from the basidiomycetes culture of *Steccherinum ochraceum* [63], *Polyporus versicolor* [64] and a microbial consortium [65].

### 3.6. Cellulase

Cellulase enzymes are the third most important enzyme for industrial uses: world-wide research has been focused on the commercial potential of cellulolytic enzymes for the commercial production of glucose feedstock from the agricultural cellulosic materials [1]. The significance of cellulose hydrolyzing thermophilic enzymes in various industries includes the production of bio-ethanol and value-added organic compounds from renewable agricultural residues [66]. Cellulose is the most abundant natural resource available globally for bioconversion into numerous products in bio-industry on a commercial scale. For efficient bioconversion a strategy of efficient saccharification using cellulolytic enzymes is required. Hardiman *et al*. [66] used the approach of thermophilc directed evolution of a thermophilic β-glucosidase. 

Cellulase is complex of three important enzymes which work synergistically owing to the crystalline and amorphous complex structure of cellulose. These enzymes, acting synergistically, hydrolyse cellulose to cello-biose, glucose and oligo-saccharides. Endoglucanase enzyme is the first one acting on amorphous cellulose fibers, attacking the glucose-polymer chain randomly, which releases small fibers consisting of free-reducing and non-reducing ends. The free-ends of the chain are then exposed to the activity of exoglucanase enzyme, which produces cellobiose. The third component of cellulase is β-glucosidase, which hydrolyses the cellobiose, producing the glucose as the final product of cellulose sacharification. 

Thermostability is an important technical property for cellulases: since the saccharification of cellulose is faster at higher temperatures, the stability of enzyme activity is necessary to be maintained for the completion of the process. Though the enzymes have been prepared using thermophilic microorganisms, these enzyme preparations are not necessarily heat-stable. The activity profile for the thermal activation and stability of cellulases derived from two *Basidiomycetes* cultures was studied by Nigam and Prabhu [67]. The results proved that the prior heat-treatment of enzyme preparation caused activation of exo- and endo-glucanase activities, and improved the stability of enzymes over a period of reaction time. Therefore, the efficiency of cellulolytic enzymes may be increased by heat-treatment, by incubating buffered enzyme preparations without cellulose or substrates prior to the saccharification process [67].

Cellulolytic enzymes have been produced by a range of microorganisms including bacteria and fungi. The studies have been performed for the biosynthesis of a high-activity preparation in high yields [68,69,70]. Researchers have cultivated microorganisms to achieve cellulases of desired quality under submerged and solid state fermentation conditions for the economical production of enzyme using waste agricultural residues [1].

### 3.7. Miscellaneous Enzymes in Biotechnology

Various enzymes other than those described above have a significant place in the list of microbial enzymes, which have established their applications in bio-industries. Lipases have been widely studied for their properties and utilization in many industries [71,72,73,74,75]. Pectinases have established their role in the fruit and juice industries [76]. Certain enzymes are specifically required in pharmaceutical industry for diagnostic kits and analytical assays [77,78,79,80]. 

Bornscheuer *et al*. [81] have currently mentioned that in all the research and developments so far in the field of biocatalysis, the researchers have contributed in three waves of outcomes. These innovations have played an important role in the establishment of current commercially successful level of bio-industries. As a result recent bioprocess-technology is capable of meeting future challenges and the requirements of conventional and modern industries, for example Trincone [82] has reviewed the options for unique enzymatic preparation of glycosides. Earlier enzymatic process were performed within the limitations of an enzyme, whereas currently with the knowledge of modern techniques, the enzyme can be engineered to be a suitable biocatalyst to meet the process requirement. Riva [83] has identified the scope of a long-term research in biocatalysis, since there are underlying problems in the shift from classical processes to bio-based processes for commercial market.

Table 1 summarizes some enzymes produced by microorganisms possessing special characteristics useful in various bio-processes. There is a tremendous scope for research and development to meet the challenges of third generation biorefeneries [83], for the production of numerous chemicals and bio-products from renewable biomasses [34]; or by the new glycoside hydrolases [82]; or new enzymes found in marine environments [84]. Although the research for the hemicellulases as important biorefining enzymes has not well established, biocatalysis for xylan processing is slowly progressing and a wide range of hemicellulases have been isolated and characterized [85]. Specifically about the biobased glycosynthesis, Trincone [82] has mentioned that the new prospects are open for the use of pentose sugars as main building blocks for engineered pentosides to be used as non-ionic surfactants or as the ingredients for prebiotic food and feed preparations. 

**Table 1:** A summarized overview of some microbial enzymes with special characteristics of industrial importance.

Enzyme	Properties	Producer Microbes-	Applications	Reference
PROTEASE (Proteolytic activity)	Acidic, Neutral, Alkaline, Thermophilic, Active in presence of inhibitory compounds	*Bacilli*; *Pseudomonas*; *Clostridium*; *Rhizopus*; *Penicillium*; *Aspergillus*	Washing Powders; Detergents; Tannery; Food Industry; Leather processing; Pharmaceuticals; Molecular Biology; Peptide synthesis	[1,3,11,12,13,14,15,16,17,18,19,34]
KERATINASE (Keratin-hydrolysing activity)	Specific Proteolytic Activity for Insoluble & Fibrous Proteins in furs, feathers, wool, hair; Thermophilic; Alkalophilic; Oxidation-Resistant	Bacteria; Actinomycetes; Fungi	Animal Feed Production; Textile Processing; Detergent Formulation; Leather Manufacturing; Medicine	[1,20,21,22,23,24,25,34]
AMYLASE (Starch-hydrolyzing activity)	Thermotolerant, Thermostable, Alkali-resistant-Exo-, endo-, de-branching, cyclodextrin-producing enzymes	*Bacillus* sp.; *Geobacillus*	Starch industry (for liquefaction); Paper, Food industry (Glucose & Maltose syrups, High Fructose Corn syrups, clarified fruit-juices); Pharmaceutical industries (Digestive aid); Brewing Industry (Starch-processing); Textile industry (Warp-sizing of fibers); Baking industry (delayed staling)	[1,26,27,28,29,30,31,32,33,34]
XYLANASE (Xylan–Pentose polymer hydrolyzing activity)	Extremophilic characteristics–Alkalophilic, Thermophilic & Thermostable	*Thermoactinomyces thalophilus*; *Bacillus* sp.; *Humicola insolens. Bispora* (acidophilic fungus)	Pentose production - Bioconversion of hemicellulose for fuel & Chemicals; Fruit-juice clarification; Improving rumen digestion; Paper industry- selective removals of xylans from kraft-pulp; Brewing industry	[1,34,35,36,37,38,39,40,41,42,43,44,45,46]
LIGNINASE (Ligninolytic-Complex-enzyme)	Oxidative properties in Lignin peroxidase, Manganese peroxidase & Laccase; Thermophilic	Basidiomycetes strains—*Steccherinum ochraceum*, *Polyporus versicolor, Panus tigrinus*	Denim washing; Bio-sensors; Bio-bleaching of Kraft-pulp; Bioremediation; Pollution-control; Treatment of recalcitrant chemicals in Textile and Industrial effluents	[1,34,47,48,49,50,51,52,53,54,55,56,57,58,59,60,61,62,63,64,65]
CELLULASE (Cellulolytic-complex enzyme)	Saccahrification of crystalline & amorphous cellulose; Thermophilic; Thermostable	Basidiomycetes strains *Polyporus* sp.; *Pleurotus* sp.; *Trichoderma* sp.; *Aspergillus* sp.	Glucose feedstock from cellulose; Bio-refinery; Bio-ethanol; Paper-pulp industry	[1,34,66,67,68,69,70]
LIPASE (Lipolytic activity)	Fat- splitting; Stereoselectivity; Racemic-Resolution activity; Solvents-resistant; Thermotolerant	Yeasts and Fungal strains-*Candida* sp., *Aspergillus* sp, *Penicillium* sp., *Rhizopus*, *Mucor*	Detergents; Dairy Industry-oils, fats, Butter, Cream, Fat-Spreads; Feed supplement; Therapeutic agent	[1,34,71,72,73,74,75]

## 4. Conclusions

Biotechnology is utilizing a wide range of enzymes synthesized on a commercial scale employing purposely screened microorganisms. Selected microorganisms have been characterized, purposely designed and optimized to produce a high-quality enzyme preparation on large scales for industrial applications. Different industries require enzymes for different purposes; hence microbial enzymes have been studied for their special characteristics applicable in various bio-processes. Recent molecular biology techniques have allowed to tailor a specific microorganism, to produce not only the high yields of an enzyme, but also enzyme with desired special characteristics such as thermostability, tolerance at high temperature and its stability in acidic or alkaline environment, and retaining the enzyme activity under severe reaction conditions such as in presence of other metals and compounds.
